# Qualitative Analysis by Experts of the Essential Elements of the Nursing Practice Environments Proposed by the TOP10 Questionnaire of Assessment of Environments in Primary Health Care

**DOI:** 10.3390/ijerph17207520

**Published:** 2020-10-15

**Authors:** José Ramón Martínez-Riera, Raúl Juárez-Vela, Miguel Ángel Díaz-Herrera, Raimunda Montejano-Lozoya, Vicente Doménech-Briz, José Vicente Benavent-Cervera, Ana Cristina Cabellos-García, Pedro Melo, Tam H. Nguyen, Vicente Gea-Caballero

**Affiliations:** 1 Departamento Enfermería Comunitaria, Medicina Preventiva y Salud Pública e Historia de la Ciencia, Universidad de Alicante, E-03080 Alicante, Spain; jr.martinez@ua.es; 2 Department of Nursing, University of La Rioja, 26006 Logroño, La Rioja, Spain; 3 Research Group BMP Idi-Paz. Hospital La- Paz, Paseo de la Castellana, 261, 28046 Madrid, Spain; 4 Primary Care Costa Ponent, Catalan Health Institute, Hospital Universitari General de Catalunya, Avda, De la Gran Vía de l´Hospitalet de Llobregat, 08908 Barcelona, Spain; 5 Hospital Universitari General de Catalunya, Carrer Pedro i Pons, 1, 08195 Sant Cugat del Vallès, Barcelona, Spain; 6 Nursing School La Fe, adscript center of Universidad de Valencia, 46026 Valencia, Spain; montejano_rai@gva.es (R.M.-L.); gea_vic@gva.es (V.G.-C.); 7 Research Group GREIACC, Health Research Institute La Fe, Avda. Fernando Abril Martorell, 106. Pabellón docente Torre H, Hospital La Fe, 46016 Valencia, Spain; anacris095@gmail.com; 8 Hospital Universitario de la Ribera, km 1, Ctra. Corbera, 46600 Alzira, Valencia, Spain; vidobri@gmail.com; 9 Department of Health Sciences, VIU Valencia International University, Calle del Pintor Sorolla 21, 46002 Valencia, Spain; josevicente.benavent@campusviu.es; 10 Hospital Universitari i Politècnic La Fe, Avda. Fernando Abril Martorell, 106. Pabellón docente Torre H, Hospital La Fe, PC, 46016 Valencia, Spain; 11 Universidade Católica Portuguesa, Institute of Health Sciences/School of Nursing (Porto)/Centre for interdisciplinary research in Health, 4200-450 Porto, Portugal; pmelo@porto.ucp.pt; 12 William F. Connell School of Nursing, Boston College, Newton, MA 02467, USA; tam.nguyen@bc.edu

**Keywords:** nurses, primary health care, community health nursing, qualitative research, working environment, consensus, quality of health care

## Abstract

*Background:* A short TOP10 scale based on the Practice Environment Scale-Nursing Work Index questionnaire measures the characteristics of nursing work environments. Positive environments result in better quality care and health outcomes. *Objective:* To identify a small number of core elements that would facilitate more effective interventions by nurse managers, and compare them with the essential elements proposed by the TOP10. *Method:* Qualitative research by a nominal group of eight experts. The content analysis was combined with descriptive data. *Results:* Ten most important items were selected and analyzed by the expert group. A high level of consensus in four items (2, 15, 20, 31) and an acceptable consensus in five items was reached (6, 11, 14, 18, 26). The tenth item in the top ten was selected from content analysis (19). The expert group agreed 90% with the elements selected as essential to the TOP10. *Conclusion:* The expert group achieved a high level of consensus that supports 90% of the essential elements of primary care settings proposed by the TOP10 questionnaire. Organizational changes implemented by managers to improve working environments must be prioritized following our results, so care delivery and health outcomes can be further improved.

## 1. Introduction

The “Magnet” concept was first applied to the field of healthcare in 1983, in a historical study [1] that proved the capability of certain hospitals in the United States of America to ‘retain’ nurses in a context where they were in scarce supply. This model of hospital achieved better outcomes for patients with a better quality of care [2]; lower mortality rates and fewer complications [3] and greater professional development [4], resulting in organizations that operate more efficiently, keeping administrative and running costs contained [5]. These better results are a consequence of a hospital organizational model that presents better conditions for providing care; according to Lake, “the nursing practice environment was defined as the organizational characteristics of a work environment that facilitate or limit the professional practice of nursing” [6].

Several questionnaires have been drawn up to measure this type of professional environment; The Practice Environment Scale of the Nursing Work Index [6] (PES-NWI). Which according to other authors [7] stands out for its methodological strength; and its 31-item version has been validated and adapted for primary (PHC) and specialized care (SC) in Spain by this authors [7].

One study [8] used another questionnaire, the Essentials of Magnetism 1 tool (EOM1), to research home health care in the USA and determine if Magnetic forces or elements in such settings were similar to those present in a hospital environment. In the hospital environment were detected as key factors: supportive nurse managers and supervisory personnel, adequate nurse staffing, working with other nurses who are clinically competent, good registered nurses–doctors relationships, nurse autonomy and accountability, continued competency in practice, adequate support services, and education. In the study’s structure and design in PHC, the authors hypothesized that if a clear relationship between the organization model and quality of care in a hospital environment existed, then the same might occur in the home care environment. They analyzed the organization of the professional practice environment and the outcomes in home health care by applying the EOM1 scale. The results demonstrated an agreement between outpatient nurses and nurses in acute medical units regarding seven out of eight Magnetic forces.

However, the large number of items present in the PES-NWI could hamper the systematic use of the questionnaire and therefore the improvement of the operational environment for nursing teams. The developer of the questionnaire suggested that a shorter version of the tool was urgently needed to facilitate data collection [6]. According to Lake, author of the PES-NWI questionnaire, “the nursing practice environment is a complex construct to conceptualize and measure” [6]; which also justifies the need to continue improving the tools available for measuring them.

Given that the professional nursing environments in PHC have been scarcely studied, and that there are various tools for evaluating them, we believe it is appropriate to consolidate the scarce evidence available from the TOP10 questionnaire, since it is the only short one that can be used in PHC. Such a reason justifies the identification of key elements within nursing environments. 

Except for the adaptation of PES-NWI to primary care in Spain [7], all the questionnaires to study nursing environments were developed for hospital care. However, a shortened version of PES-NWI has recently been published, called TOP10 [9], with a predictive power of 90.7% in relation to PES-NWI. To build the TOP10, care nurses and managers selected the 10 items that they considered essential elements of environments that would allow nurses to deliver the highest quality care to their patients. The weakness of any (or several) of these factors would inevitably lead to a decline in the quality of nursing care.

These essential elements of the nursing professional work environments that the nurses themselves identified as key to being able to offer quality care in the community setting are structured in three dimensions: leadership and participation in the management of the center; fundamentals of nursing and interprofessional relations; and adequate resources [9].

Another study in the Canary Islands [10] asked nurses in the public health system what elements of the settings were most conducive to providing quality care, using the PES-NWI questionnaire. The results presented a list of 12 elements considered essential. The 10 elements of the TOP10, published later, are included in the 12 identified by the nurses of the Canary Islands.

As we have seen, the record shows strong evidence that improving the quality of hospital nursing environments has a direct impact on people’s care, health outcomes, and patient satisfaction [2,3,4,5]; and recent studies in community settings appear to strengthen that evidence as well [9,10]. However, the current climate of economic disinvestment in primary care as a result of austerity policies, and the gradual encroachment of private healthcare providers [11] further endorses the need to facilitate the provision of quality nursing care in the most cost-effective manner [12].

For such reason, we decided to identify the essential items within the PES-NWI by obtaining the opinion from experts in PHC. The objective of this study was to identify a small number of core elements that would facilitate more effective interventions by nurse managers, and compare them with the essential elements proposed by the TOP10. This expert analysis could offer new approaches to improve the TOP10 short questionnaire, improving its predictive power and psychometric properties.

## 2. Materials and Methods 

### 2.1. Design

We carried out a qualitative study (2015): a descriptive recount of responses [13] (individual activity, phase 1) combined with a second phase consisting of a nominal group of experts (group activity) as an approach to achieve consensus, with discussion and content analysis. 

### 2.2. Sample

We identified an initial purposive sample of experts (including those meeting ad hoc criteria such as employment at a university, primary health center, research department, and/or managers-administrators in PHC) and supplemented the initial group of experts with others identified by ‘snowball’ sampling and who was approached by mail. The nominal group participants and the main researcher were unknown to each other. Our final sample included 8 participants (100% participation rate, 0% refused) (Table 1). 

### 2.3. Process

The recount of individual results (with the PES-NWI tool, 31-item Spanish version) was carried out before and after the development of the nominal group (an audio conference for two hours at home, with a moderator—male, Ph.D. student, PHC nurse and Lecturer, interested in nursing work environments- and a participant—female, Ph.D. student, Lecturer) and with a moderator fulfilling an explicit mediation role (no one was present). Subsequently, the count was made again (after the group discussion), and finally, the analysis content was done, for discrimination of doubtful items. The doubts were finally consulted by mail.

We aimed to complement the evidence obtained from each methodology (recount/nominal group-content analysis) [14] and proceed to integrate it using a triangulation approach. The data was discussed until thematic saturation was obtained.

The focus group activity was recorded (audio recording) and the content was transcribed verbatim. 

### 2.4. Data Analysis

The content was analyzed with the aid of QSR NVivo 10 software (QSRInternational, Burlington, MA, USA) by the two main researchers. A thematic analysis of the group discussion was conducted, building thematic categories and, identifying the relevant information nodes to understand the key elements in the working environments.

Socio-demographic data as well as descriptive analyses of the responses (frequencies and percentages) were conducted with SPSS v20 software (SPSS Inc., Chicago, IL, USA). The analysis was complemented by the field notes collated by the focus group moderator (principal researcher) and participant (assistant).

For convenience, the level of consensus among the experts was defined as using previously established criteria [14] (Table 2), understanding by consensus the level of agreement or disagreement among experts in the selection of the essential elements.

### 2.5. Ethical Considerations

The identity of the participants was kept anonymous. All recordings and transcriptions were kept in institutional, password-protected computers. Data were anonymized and protected according to Spanish law (organic law 15/1999), and its European equivalent, 95/46/EC. 

The protocol was approved by the Ethics Committee in Xàtiva/Ontinyent Department (Comunitat Valenciana, Spain) (#2522014). All participants were informed about the aims of the study and their informed consent was obtained before their participation. The authors declare no conflict of interest or funding. 

## 3. Results

63% of the participants were women; 75% were over 40 years of age (average 47.5, standard deviation 8.29); 75% had more than 10 years of experience in PHC, with 5–10 years for the rest. All participants had experience as nursing lecturers and researchers. 87.5% had conducted or led technical projects in community nursing. Finally, 87.5% belonged or had belonged to a scientific nursing association.

### 3.1. Phase 1

Table 3 shows the results of phase 1 (recounts of individual results before and after developing the nominal group). There was an uncertain level of consensus on 5 items, requiring a third count, which did not resolve the doubts. Items 13 (Nursing care is based on a nursing model, more than on a biomedical model) and 19 (Nurses in the center have appropriate clinical competence) were each chosen three times (uncertain level of consensus), the rest were removed. At this stage, an agreement was reached on 9 of 10 items.

### 3.2. Phase 2

In phase 2 (nominal group), the key research questions for the focus group were the following, which corresponds to the nodes of information we created (two data coders):

1. Do you think this item or others may not be relevant in reaching excellent nursing care in your professional work environment?
2. Do you think this item is fundamental in guaranteeing excellent nursing care in your professional work environment?
3. Do you think this item is strongly related to another/other item(s) that we have already discussed or those we will discuss subsequently?

Furthermore, a fourth content category deemed to be complementary and inclusive was developed following the data analysis. This fourth category included two items (item 3—professional development, and item 6—professional career) that had previously generated doubts:

Might this item generate doubts of understanding with reference to what it aims to measure?

#### Content Analysis

The expressions recovered by nodes of information were the following: 

Category 1: Non-relevant items. 7 items (items 16, 21, 22, 23, 24, 27 and 30) were discarded by the focus group as they did not receive any votes: *(…) if no-one has selected them, it is because they appear to be less important than the others* (E3) (whole group); *(…) It is proposed that… in any case we can leave them to the end, if the rest of the items aren’t agreed on, we could argue over why they haven’t been chosen, that could be interesting* (E5); *(…) Focus on the rest and discuss it afterward as may enhance the debate* (E6).

Categories 2, 3, and 4: Categories 2 and 3 were addressed together given that during the focus group session there were comments and suggestions made about the similar content of the items. The grouping was carried out following the order of appearance of the dimensions in the questionnaire, with minor modifications as a result of suggestions made by the expert group.

#### 3.2.1. Dimension 1: Nurse Participation in Center Affairs

Item 2—Opportunity for staff nurses to participate in policy decisions. This dimension was approached as a category 3 item, alongside item 1 (Staff nurses are involved in the internal governance of the hospital): *(…) in my opinion, it’s really important to give your opinion, to participate, but I think it has been forgotten about a bit* (E1); *For me, between 1 and 2, I’d go for 2 (…) not at a center level, but rather at management level in primary healthcare that would encompass both aspects* (E3); *in my opinion, 2 seems more important than 1* (E7); *yes, yes, 2 seems more important (…)* (all group).

Therefore, item 2 was prioritized over item 1, as the experts considered that although the items were related, item 2 had to be given greater importance as political decisions were considered to be more likely to contribute to care improvements. 

Item 6—Career development/clinical ladder opportunity. This item was approached as category 3, along with item 3 (There are many professional development opportunities for nursing staff). However, as there was no clear conceptual difference between development and career, a category 4 content was created with these two items: *(...) Personal professional development is something you seek out yourself, you do it yourself, the organization can’t force it upon you, it depends on each of us* (E2); *I understand items 3 and 6 to be equivalents, it has been exclusively linked to seniority, based on retributive factors* (E4); *The end I went for item 6 because what’s the point in being a well-developed professional if there’s no way of climbing the professional ladder* (E3)*; The Healthcare Providers Laws and Regulations (Ley de Ordenación de las Profesiones Sanitarias, 2003) establishes that organizations must encourage its professionals to undertake professional development, and it has an obligation to develop a professional career system.* (E4)*; yes number 6, obviously (...)* (E8, all group). 

The possibility of developing a clinical career was chosen before professional development.

#### 3.2.2. Dimension 2: Nursing Foundation for Quality of Care

Item 11—There is an active quality assurance program. Considered unanimously by the group as a crucial item, and thus included as Category 2 item: *Really, really, very important* (E2); *(...) I didn’t prioritize it, but the truth is that it is very important...but yes, I do think it is important* (E5).

Item 14—Patient care assignments that foster continuity of care (e.g., the same nurse looks after the patient for the whole time). Content Category 2. The majority of the group considered that this item would be a requisite or axiom, and its absence would complicate the provision of personalized care. For example, primary healthcare work allocated by quotas demands that this item is present, as it would otherwise be unfeasible to provide quality care: *It’s a necessary condition, it’s essential* (E3); *I can’t conceive working without it, it’s not that it is a priority, it’s that without it you cannot provide personalized care, you cannot focus the care, and I simply can’t conceive working without It (...)* (E5).

Item 15—A clear philosophy of nursing that permeates the patient care environment. Information content category 3, when debated alongside item 13 (Nursing care is based on a nursing model, rather than a medical model): *(...) 15 is broader than 13, and not so much the doctor or nurse’s role as a profession, and it puts the focus on care* (E1); *I think 15 is a priority and it is essential, a philosophy* (E3); *I had chosen 13, but looking at it now, I think I’d go for 15* (E7).

Item 18—Active staff development or continuing education programs for nurses. Content category 2. Virtually, all the participants considered it very important. Even those who did not prioritize it stated that if they could choose another item, they would choose this one. In general, the comments were unanimous, for example: *(...) it’s very important and I prioritized it* (all group).

Item 19—Working with clinically competent nurses. Content category 2. The analysis of item 19 was also important given that an insufficient consensus was generated in the previous phase. The evaluation provided by the group components may be considered key: *I can’t imagine quality without it* (E2); *It is a requirement* (E3); *It is not a requirement, but it’s a condition for excellence* (E7); *We are asked for tons of competences, and all of them are linked to being able to offer quality care* (E8).

Only one participant was doubtful about this item: (...) That’s a desire, isn’t it (E4); We assume they are competent. Don’t we? (E4).

#### 3.2.3. Dimension 3: Nurse Manager Ability, Leadership, and Support of Nurses

The whole dimension is considered to be as a content category 3. 

Item 20—The manager (supervisor/coordinator) is a good manager and leader. Chosen by all the focus group participants, who agreed that a good manager and leader fulfilled the characteristics encompassed in the rest of the items (...item 20 compiles 21, 22, 23 and 24... the qualities of good manager...). They were:

Item 21—A nurse manager who backs up the nursing staff in decision-making, even if the conflict is with a physician.

Item 22—The supervisor uses mistakes as learning opportunities, not as criticisms.

Item 23—A supervisory staff that is supportive of the nurses.

Item 24—Praise and recognition for a job well done.

#### 3.2.4. Dimension 4: Staffing and Resource Adequacy

The group came together to evaluate this category, due to its limited content (4 items). Item 27 had not been preselected. 

Item 25—Enough staff to get the work done.

Item 26—Enough qualified (registered-nurses) to provide quality patient care.

Item 28—Enough time and opportunity to discuss patient care problems with other nurses.

The references were mostly focused on the importance of nursing resources. When the scoring of the recount was considered, item 26 generated an acceptable consensus and higher overall score than the rest of the items: *(...) I wavered between 25 and 26...if it is of employees or nurses* (E1); *I prioritize nursing resources more...that’s obvious* (E3); *It’s necessary to have enough staff; and we need them to be competent* (E8).

#### 3.2.5. Dimension 5: Nurse–Physician Relationships

This dimension continues on from a similar process to the previous dimension. Items 29 and 31 were preselected.

Item 29—A lot of teamwork between nurses and physicians.

Item 31—Collaboration -joint practice- between nurses and physicians.

The reference to the data count showed conclusive outcomes, with a high level of consensus (chosen by ~75% of experts after the group discussion) for item 31; however, no participants selected item 29; they said: *I went for 31 straight away...I had no doubt at all about it* (E4)*; I preferred cooperation; I had no doubt about it, 31* (E5)*; I had no doubt about it, 31 as well* (E6).

Global results: Finally, after analyzing the overall outcomes from phases 1 and 2, the opinion of the 8 experts in PHC was that the 10 most important elements that shape the environment for professional nursing practice were (Table 4). 

## 4. Discussion

Our study aimed to synthesize and prioritize ten key items within the 31 original items featured in the PES-NWI, as selected by consensus by a group of experts (PHC). Subsequently, we try to compare this selection of ten essential elements with those proposed in the TOP10 questionnaire [10]. We have used a comprehensive methodology and our results replicate and complement those achieved previously. 

The PES-NWI questionnaire was used recently in Spain to measure the work environments for nurses in PHC [7,15,16,17,18]. It allowed the identification of specific areas which were scored positively or negatively, therefore providing a development plan for each unit or center. The high scores in the questionnaire highlighted areas in the working environment that was already well developed by managers to facilitate the professional activity of nurses. However, it is necessary to determine which are the essential elements on which management teams should intervene as a priority to improve results, since we know that better environments produce better health outcomes [3,5,9,10].

Concerning to improving care management in the Health Plan of the Catalan Institute of Health 2011–2015 [19], our outcomes confirm the importance of factors such as training, nurse–doctor relationships, clinical competence, management, and sufficient human resources, with the participation of professionals in the model of care and management. With regard to the nurse–doctor professional relationship, a study [18] suggested that relationships were better in PHC compared to SC. The least valued factor was staff suitability, which we proposed as a fundamental element when considering clinical and process outcomes such as mortality, healthcare-associated infections, and complications, etc. [2,3,4].

If we compare the choice of key elements of the professional practice environments in the TOP10 [9], we observe that it is highly coincidental, in 90% of the elements (9 of 10 elements) (Table 5). 

These results are significant since we consider that a coincidence power of 90% is very high and would confirm, at least 9 of the 10 elements, that are really essential to improve the quality of care. According to the expert nurses, it is more important that nurses have the opportunity for adequate career development, to improve as a professional and provide better care. Expert nurses did not prioritize an element related to human resources. In the group discussion, the experts believed that the existence of enough nurses is an essential requirement for care, being not so important the rest of professionals who are not nurses. 

On the other hand, the divergence in 1 of the 10 items generates the need to perform a psychometric test of the TOP10, replacing item 25 with item 6. It would be necessary to know if the predictive power of the TOP10 with this modification improves in relation to the complete PES-NWI questionnaire. 

The results of the study are also consistent with those of a study conducted in primary care in the Canarian Islands [10]. In this study, the nurses proposed 12 essential elements, including all 10 proposed by our experts (100%). 

In comparison with questionnaire EOM 1 [20] in the USA, and in spite of its antiquity, which did prioritize 10 characteristics, we observed similarities: ‘active program of quality’, ‘an existing philosophy on nurse care’, ‘development of training program’, ‘professional clinical competence’, ‘appropriate professional cooperative nurse–doctor relationships’, ‘enough nursing staff to achieve quality care’ and ‘quality supervision’. Therefore, this would represent an agreement of 7 essential factors with the EOM 1 questionnaire. In addition, this proposal fits perfectly with the results proposed by the group of experts, who proposed a professional development plan, and only the element of sufficient nurses. Item 20 (The supervisor/coordinator is a good manager and leader), is implicit in the PES-NWI questionnaire. These latter conclusions were obtained in hospital environments. However, Mensik used the EOM1 tool in home care in the USA [8] and obtained similar results in at least 5 factors: ‘training’, ‘clinical competence’, ‘supervision as a leader’, ‘enough nursing staff’ and ‘nurse–doctor relationships’.

In another study based on hospital environments in Belgium [21] it is explored the organizational characteristics that encouraged the retention of nurses. He concluded that hospitals with a ‘flat’ hierarchical structure (i.e. management accessible to clinical nurses) in which nurses participated in management issues, structured training organizations, and opportunities to develop a professional career in the hospital, fostered higher levels of satisfaction in the workplace with an increase in loyalty towards the organization. 

This study agrees with the outcomes obtained in our expert nominal group study, who emphasized ‘competence and autonomy’, ‘educational aspects within the organization’, ‘participative management developed by good leaders’, ‘the interaction between professionals’, as well as the chance to gain a career promotion within the organization; and agree too with the essentials elements proposed in TOP10 tool [9]. Similar coincidences could suggest that essential items for improving our working environments do exist. We consider that enhancing the professional development of nurses contributes to improved outcomes, professional satisfaction and commitment to the health organization. 

The author of the questionnaire PES-NWI [22] suggests that given that these items are variable, they can be modified so they become optimum and seek excellence. In this sense, those who assessed PHC environments in Madrid (Spain), agreed with the author [18], concurring that awareness of the working environment factors allows to optimize the provision of care and to improve healthcare outcomes. 

In a second validation of the PES-NWI questionnaire in Spain [23], highlighted the importance of using these questionnaires and considering the environment to improve patients’ health. Similarly, another study [24] proposed that gaining a better environment facilitates the incorporation of clinical evidence into work. By focusing innovation strategies and improvement initiatives on the elements highlighted as essential in our study, organizations could improve service delivery and business performance as well as optimal public health results. Such a perspective would offer an optimal response to ongoing privatization negatively affecting many structural determinants of citizen health [11], whilst containing costs and improve working conditions [5]. 

For all these reasons, innovation and investment in PHC must contribute to improving nursing participation in management, the growth in the number of nurses on health teams (since the number of nurses per inhabitant in Spain is one of the lowest in Europe and the world [25]) and the incorporation of new professional figures (especially specialist nurses and advanced practice nurses). 

Increasing the nursing ratios in Spain should be a priority. All the studies of nursing environments in Spain, both, in hospital and PHC settings, show that the perception of nurses regarding human resources is low. This is confirmed by the figures provided by various studies, which show that the shortage of nurses can be up to 37.6% lower than the European average [26]. This fact is very serious, since in PHC the workload in Spain prioritizes more clinical care, with a deficit of resources allocated to community care. And this is linked with professional development. The shortage of specialist and advanced practice nurses at PHC is also a consequence of the lack of nurses and their poor professional development; the contribution of these figures can be very valuable for community health and for chronicity care [27]. This health care implemented by advanced practice nurses is also of higher quality, safety, and better cost-effectiveness [27].

Therefore, work environments are a strategic element that should facilitate the work of nurses in the community. Because, as some authors suggest, the involvement of nurses as community health agents integrated into the health team is vital for community health [28]. 

Limitations: The main limitation of the present study has been the fact of carrying out a qualitative study in a specific geographical, cultural and socio-demographic environment of 1 country (Spain). Therefore, the condition of experts in primary care is limited to a deep knowledge of the Spanish health system and its PHC model. Other geographical contexts could have different realities and differ in the results. However, the results have been triangulated and the high level of coincidence with international precedents allows us to have confidence in the results obtained. Besides, the scarcity of studies on professional settings at PHC is itself a limitation for obtaining information.

## 5. Conclusions

We believe that determining the existence of fundamental items that show stability in different contexts and cultures may contribute to improved nursing work environments. Even if the 31 factors of the original tool were assumed to be equally important, we would consider it too difficult to act efficiently on each of them to optimize the workplace environment. The expert group achieved a high level of consensus that supports 90% of the essential elements of primary care settings proposed by the TOP10 questionnaire. We recommend that interventions should focus instead on the elements identified in our study as fundamental. Organizational changes implemented by managers to improve working environments must be prioritized following our results, so care delivery and health outcomes can be further improved.

## Figures and Tables

**Table 1:** Profile of experts participating in nominal group.

Expert Identity	Education	Experience (years)	Current Post	Other PHC ^1^ Activities	Health Technician	Manager	Research	Scientific Association
E1	RN ^2^	7	FNP ^1^	Yes	Yes	No	Yes	Yes
E2	RN ^2^ OD ^3^	7	Deputy Charge Nurse	Yes	No	Yes	Yes	Yes
E3	RN ^2^ OD ^3^ PhD ^4^	>10	Associate Professor	Yes	Yes	Yes	Yes	Yes
E4	RN ^2^ OD ^3^	6	FNP ^1^	Yes	Yes	Yes	Yes	Yes
E5	RN ^2^ OD ^3^	9	Associate Professor	Yes	Yes	Yes	Yes	No
E6	RN ^2^ MSc ^5^	>10	FNP ^1^ Lecturer	Yes	Yes	No	Yes	Yes
E7	RN ^2^	>10	Health Services Director	Yes	Yes	Yes	Yes	Yes
E8	RN ^2^ MSc ^5^	>10	FNP ^1^ Lecturer	Yes	Yes	No	Yes	Yes

^1^ FNP: Family Nurse Practitioner; ^2^ RN: Registered Nurse (Graduate in Nursing); ^3^ OD: Other Degree; ^4^ Ph.D.: Doctor; ^5^ MSc: Master of Science.

**Table 2:** Level of consensus among experts.

High Level of Consensus	Item Selected by 6 or More Experts (≥75%)
Acceptable level of consensus	Item selected by 4 or 5 experts (50–74%).
Uncertain level of consensus	Item selected by 3 experts (at least 37.5%). In this case, we opted for assessing the qualitative analysis and avoid the recount technique.
Very low level of consensus	Item selected by 1 or 2 experts (25% or less). These items were analysed using a transcription analysis.
Null consensus	Items not selected as important by any expert. These items were removed from the group discussion straight away (7 of 31 items, 22.5% of the total).

**Table 3:** Main items with the experts’ count stage results, pre and post the focus group.

Item Heading	PRE ^1^ (N/%)	POST ^2^ (N/%)
2. Opportunity for staff nurses to participate in policy decisions.	6 (75%)	8 (100%)
6. Career development/clinical ladder opportunity.	4 (50%)	4 (50%)
11. There is an active quality assurance program.	5 (62.5%)	4 (50%)
14. Patient care assignments that foster continuity of care (e.g., the same nurse looks after the patient for the whole time).	4 (50%)	5 (62.5%)
15. A clear philosophy of nursing that pervades the patient care environment.	5 (62.5%)	8 (100%)
18. Active staff development or continuing education programs for nurses.	4 (50%)	4 (50%)
20. A nurse manager (supervisor/coordinator) is a good manager and leader.	5 (62.5%)	8 (100%)
26. There are enough qualified nurses to provide quality patient care.	3 (37.5%)	4 (50%)
31. Collaboration (joint practice) between nurses and physicians	4 (50%)	6 (75%)

^1^ PRE: pre-nominal group. ^2^ POST: post-nominal group.

**Table 4:** 10 main elements in the PES-NWI ^1^ questionnaire as selected by experts.

Dimension	*N* of Items by Importance	Items
1 Nurse participation in center affairs	2	2 Opportunity for staff nurses to participate in policy decisions. 6 Career development/clinical ladder opportunity.
2 Nursing foundation for quality of care	5	11 An active quality assurance program. 14 Patient care assignments that foster continuity of care (e.g., the same nurse looks after the patient for the whole time). 15 A clear philosophy of nursing that pervades the patient care environment. 18 Active staff development or continuing education programs for nurses. 19 Working with nurses who are clinically competent.
3 Nurse manager ability, leadership and support of nurses	1	20 A nurse manager (supervisor/coordinator) is a good manager and leader.
4 Staffing and resource adequacy	1	26 There are enough qualified nurses to provide quality patient care.
5 Nurse-physician relationships	1	31 Collaboration (joint practice) between nurses and physicians

^1^ PES-NWI: Practice Environment Scale of the Nursing Work Index.

**Table 5:** Differences in the elements selected between expert nurses and those proposed in the TOP10.

Items Selected by Experts (Extracted of PES-NWI)	Items TOP10 (Extracted of PES-NWI)
2 Opportunity for staff nurses to participate in policy decisions. 6 Career development/clinical ladder opportunity. * 11 An active quality assurance program. 14 Patient care assignments that foster continuity of care (e.g., the same nurse looks after the patient for the whole time). 15 A clear philosophy of nursing that pervades the patient care environment. 18 Active staff development or continuing education programs for nurses. 19 Working with nurses who are clinically competent. 20 A nurse manager (supervisor/coordinator) is a good manager and leader. 26 There are enough qualified nurses to provide quality patient care. 31 Collaboration (joint practice) between nurses and physicians	2 Opportunity for staff nurses to participate in policy decisions. 11 An active quality assurance program. 14 Patient care assignments that foster continuity of care (e.g., the same nurse looks after the patient for the whole time). 15 A clear philosophy of nursing that pervades the patient care environment. 18 Active staff development or continuing education programs for nurses. 19 Working with nurses who are clinically competent. 20 A nurse manager (supervisor/coordinator) is a good manager and leader. 25 There are enough health workers to provide quality patient care. * 26 There are enough qualified nurses to provide quality patient care. 31 Collaboration (joint practice) between nurses and physicians

* Items in which the two studies differ are in bold.
